# Development of a novel medium throughput flow-cytometry based micro-neutralisation test for SARS-CoV-2 with applications in clinical vaccine trials and antibody screening

**DOI:** 10.1371/journal.pone.0294262

**Published:** 2023-11-30

**Authors:** Sophie O’Reilly, Grace Kenny, Tamara Alrawahneh, Nathan Francois, Lili Gu, Matthew Angeliadis, Valentin de Masson d’Autume, Alejandro Garcia Leon, Eoin R. Feeney, Obada Yousif, Aoife Cotter, Eoghan de Barra, Mary Horgan, Patrick W. G. Mallon, Virginie Gautier

**Affiliations:** 1 Centre for Experimental Pathogen Host Research (CEPHR), University College Dublin, Belfield, Dublin, Ireland; 2 School of Medicine, University College Dublin, Belfield, Dublin, Ireland; 3 Department of Infectious Diseases, St Vincent’s University Hospital, Elm Park, Dublin, Ireland; 4 Endocrinology Department, Wexford General Hospital, Carricklawn, Wexford, Ireland; 5 Department of Infectious Diseases, Mater Misericordiae University Hospital, Eccles St, Dublin, Ireland; 6 Department of Infectious Diseases, Beaumont Hospital, Beaumont, Dublin, Ireland; 7 Department of International Health and Tropical Medicine, Royal College of Surgeons in Ireland, Dublin, Ireland; 8 Department of Infectious Diseases, Cork University Hospital, Wilton, Cork, Ireland; 9 Conway Institute of Biomedical and Biomolecular Research, University College Dublin, Belfield, Dublin 4, Ireland; University of Bologna / Romagna Local Health Authority, ITALY

## Abstract

Quantifying neutralising capacity of circulating SARS-COV-2 antibodies is critical in evaluating protective humoral immune responses generated post-infection/post-vaccination. Here we describe a novel medium-throughput flow cytometry-based micro-neutralisation test to evaluate Neutralising Antibody (NAb) responses against live SARS-CoV-2 Wild Type and Variants of Concern (VOC) in convalescent/vaccinated populations. Flow Cytometry-Based Micro-Neutralisation Test (Micro-NT) was performed in 96-well plates using clinical isolates WT-B, WT-B.1.177.18 and/or VOCs Beta and Omicron. Plasma samples (All Ireland Infectious Diseases (AIID) Cohort) were serially diluted (8 points, half-log) from 1:20 and pre-incubated with SARS-CoV-2 (1h, 37°C). Virus-plasma mixture were added onto Vero E6 or Vero E6/TMPRSS2 cells for 18h. Percentage infected cells was analysed by automated flow cytometry following trypsinisation, fixation and SARS-CoV-2 Nucleoprotein intracellular staining. Half-maximal Neutralisation Titres (NT50) were determined using non-linear regression. Our assay was compared to Plaque Reduction Neutralisation Test (PRNT) and validated against the First WHO International Standard for anti-SARS-CoV-2 immunoglobulin. Both Micro-NT and PRNT achieved comparable NT50 values. Further validation showed adequate correlation with PRNT using a panel of secondary standards of clinical convalescent and vaccinated plasma samples. We found the assay to be reproducible through measuring both repeatability and intermediate precision. Screening 190 convalescent samples and 11 COVID-19 naive controls (AIID cohort) we demonstrated that Micro-NT has broad dynamic range differentiating NT50s <1/20 to >1/5000. We could also characterise immune-escape VOC Beta and Omicron BA.5, achieving fold-reductions in neutralising capacity similar to those published. Our flow cytometry-based Micro-NT is a robust and reliable assay to quantify NAb titres, and has been selected as an endpoint in clinical trials.

## Introduction

SARS-CoV-2 is the viral agent responsible for the Coronavirus Infectious Disease 2019 (COVID-19) pandemic [1]. The disease was given pandemic status by the World Health Organisation in March 2020, and as of September 2023, there has been 676 million cases, and 6.8 million deaths [2].

Both T-cell and humoral immune responses are required for protection from COVID-19. Humoral immunity relies on B cell exposure to SARS-CoV-2 antigens, which triggers their proliferation into antibody secreting plasma cells [3]. Following infection, antibodies are produced against SARS-CoV-2 viral proteins, predominantly the Spike (S) and the Nucleocapsid Protein (NP) [4,5]. Neutralising antibodies (NAbs) are a subset of SARS-CoV-2 antibodies that prevent viral entry, through either direct blocking of virus binding to the host cell receptor, or preventing conformational changes required for membrane fusion. SARS-CoV-2 NAbs target the S protein, making it the preferred COVID-19 vaccine candidate [6,7]. The S protein is comprised of trimeric S1/S2 heterodimers. S1, harbouring an N-terminal domain and a Receptor Binding Domain (RBD), interacts with the host cell through binding of the RBD to the angiotensin-converting enzyme 2 (ACE-2) receptor. Following S1/S2 cleavage by host cell proteases including furin, S2’ is cleaved by TMPRSS2 or Cathepsin-L mediating membrane fusion and cell entry [8]. The most potent NAbs target the RBD as these directly compete with ACE-2 for binding. Mutations in this site are often associated with immune escape [9]. Non-RBD sites are more evolutionarily conserved, so NAbs targeting these sites can often maintain efficacy against SARS-CoV-2 variants as well as display cross-reactivity with other sarbecoviruses [10].

An effective antibody response provides protection against COVID-19. ‘The Protective Neutralisation Classification Model’ described by Khoury and colleagues [6] suggests that the protective neutralisation titre (reducing risk of infection by 50%) is 20% of the mean neutralisation titre of a convalescent cohort. They have found this to strongly predict protective immunity (against symptomatic disease) elicited by COVID-19 vaccine trials, while achieving a titre of only 3% of the mean is sufficient to reduce risk of severe disease by 50%.

Not all S-targeting antibodies are neutralising. Post-infection or post-vaccination, a polyclonal antibody population is produced, targeting sites along the S protein [11]. Some may only bind, but not have any neutralising capacity due to their site of action. Others may offer protection against Wild-type (WT) SARS-CoV-2, the strain against which the vaccine S was originally modelled, but not against immune-escape Variants of Concern (VOC), including Beta which was the first SARS-CoV-2 Variant to display moderate immune escape, and Omicron, which far exceeded the escape capacity of previous variants, due to large numbers of amino acid mutations in key antibody binding sites in the RBD [12]. For this reason, an antibody titre, the measure of total anti-SARS-CoV-2 IgGs against a certain target present in a sample [13], is not sufficient to infer a protective immune response. Instead, the functional capacity of an antibody population can be determined using a neutralisation test.

Neutralisation tests, used to measure the capacity of a monoclonal antibody or plasma/serum to inhibit viral infection of susceptible cells, have proved valuable in elucidating SARS-CoV-2 antibody responses over time [14], in convalescent versus vaccinated individuals [15], and against SARS-CoV-2 VOCs [16,17]. This information is critical to forming effective public health strategies, from understanding when vaccine-induced protection wanes in different cohorts to devise booster strategies [18,19], to identifying plasma donors for convalescent therapy [20,21], to rapid identification of new VOCs that escape pre-existing immunity [22,23].

The original gold standard viral neutralisation assay is the Plaque Reduction Neutralisation Test (PRNT) [24]. Here, live SARS-CoV-2 is co-incubated with serially diluted antibodies to facilitate neutralisation, and the virus is then used to infect a monolayer of cells. Over days, infected cells will display viral cytopathic effects (CPE) and die, leaving visible plaques in the monolayer that can be quantified to determine the reduction in infectious titre associated with the dilution factor of antibodies. However, this technique has several limitations which alternative assays have been developed to address. These include the large surface area for plaque formation requiring large wells, typically 6- or 12-well, which limits the throughput. The time to develop visual plaques can be up to 5 days for SARS-CoV-2. Furthermore, plaque counting is often done by eye, and even with automated software can be error-prone and subjective.

One significant limitation of PRNT, is the use of live SARS-CoV-2. This means the assays must be carried out in Containment Level 3 facilities, by highly trained staff. Pseudovirus assays, where a viral backbone from vesicular stomatitis virus (VSV) or lentivirus, is engineered to express the SARS-CoV-2 S protein, have become popular choice as they only require Containment Level 2 facilities [25]. However, they focus on the impact of S protein independently of other SARS-CoV-2 proteins present at the viral membrane and typically only measure viral entry, while live-virus neutralisation assays can monitor several rounds of replication. As the virus can spread cell-to-cell post-entry, it may evade neutralisation, thus explaining the lower neutralisation titres observed in such assays compared to pseudovirus assay [26]. Surrogate Viral Neutralisation Tests or competitive immunoassays where antibodies prevent interaction between recombinant viral Spike protein and ACE-2 receptor *in vitro* have been proposed as a user-friendly method of inferring neutralisation capacity in the absence of virus, however they have shown poor correlation with PRNT [27].

For those with facilities to work with live virus, extensive effort has instead been put towards the development of live-virus micro-neutralisation assays, working on the same principle as PRNT, firstly facilitating neutralisation with a co-incubation of virus and antibodies, followed by determining the effectiveness of neutralisation through infection of susceptible cells. The key difference in these assays are the format (96-well plates) and the endpoints (Table 1). Rather than wait for visible plaques, micro-foci can be detected in the monolayer after only 18h infection by staining of viral antigens [24,28]. Alternatively, recombinant SARS-CoV-2 virus expressing Green Fluorescent Protein can be detected directly without the need for further processing [28]. Another approach is to detect viral genetic material in cell lysates through RT-qPCR following RNA extraction [29,30] or to quantify replicating virus (viral load) by enzyme-linked immunosorbent assay (ELISA) [31]. Other assays score the presence or absence of CPE under a microscope [32,33] which can be revealed using a cell imager [34], or using colorimetric cellular dyes [32,35]. All these assays have the advantage of being medium to high throughput and suitable for 96-well plates, while some are also rapid compared to PRNT. These micro-neutralisation assays, so called for their use of microplates to improve throughput, are now also considered gold standard assays for measuring neuralisation capacity. Table 1 provides a comparison of a non-exhaustive list of these assays.

**Table 1:** Comparison of micro-neutralisation tests.

Micro-Neutralisation Assays	Assay Endpoint	Infection Time	Viral/Cellular Target	Reference
**Flow Cytometry Based Micro-NT**	Flow Cytometry	18h	Intracellular SARS-CoV-2 NP	(This publication)
**ELISA-based virus**	Enzyme-linked immunosorbent assay (ELISA)	24h	Intracellular SARS-CoV-2 NP	[31]
**RT-qPCR-based virus neutralisation assay**	RT-qPCR	24h	Intracellular SARS-CoV-2 genomic RNA	[29,30]
**Microneutralization assay (MNA)/** **Focus reduction neutralization test (FRNT)**	Infected foci measured by computer-controlled imagers	22-24h	Cell-membrane associated SARS-CoV-2 Spike	[24,28]
**Fluorescence-based viral neutralisation assay**	Automated cell imaging	16h	mNeonGreen expressing recombinant SARS-CoV-2	[36]
**mNeonGreen-based FRNT (FRNT-mNG)**	Fluorescent ELI-SPOT	24-30h	mNeonGreen expressing recombinant SARS-CoV-2	[28]
**Virus Neutralisation Test (VNT)**	CPE observed under light microscope	4–5 days	CPE	[32,33]
**Colorimetric cytopathic effect-based microneutralization assay**	Optical density of cellular lysate	3 days	CPE	[32]
**High-Content Fluorescent live SARS-CoV-2 neutralization assay**	Nuclei counts using IN CELL analyser	3 days	CPE proxy	[34]
**Micro-neutralisation Assay**	Cell Viability measured by photometer	48h	CPE	[35]

Micro-NTs are conducted in medium-throughput formats, typically 96-well plates. Following neutralisation of live SARS-CoV-2 by serial dilutions of plasma/serum, neutralisation capacity of each dilution is determined by corresponding infection levels compared to controls. The primary difference is the endpoint used to quantify infection, which may measure viral production directly, or viral cytopathic effects (CPE).

Here we provide an alternative endpoint for a SARS-CoV-2 micro-neutralisation assay, utilising flow-cytometry to quantify the percentage of cells infected following intracellular staining of SARS-CoV-2 nucleocapsid protein at 18h post-infection. This novel, medium-throughput approach utilises a 96-well plate format enabling parallel processing of 3 plasma samples per plate across an 8-fold serial dilution.

This novel, live SARS-CoV-2, flow-cytometry based micro-neutralisation test (Micro-NT) was calibrated and validated against PRNT using the First WHO International Standard for anti-SARS-CoV-2 immunoglobulin, and a panel of secondary standards made of pooled COVID-19 convalescent and vaccinated plasma samples with high, medium and low neutralisation capacities. To further validate this platform, we confirmed good inter- and intra-assay reproducibility, followed by the capacity to generate a broad dynamic range of NT50 values when screening a large COVID-19 cohort. We also examined the capacity to identify and characterise immune escape variants.

## Results

**Fig 1:**
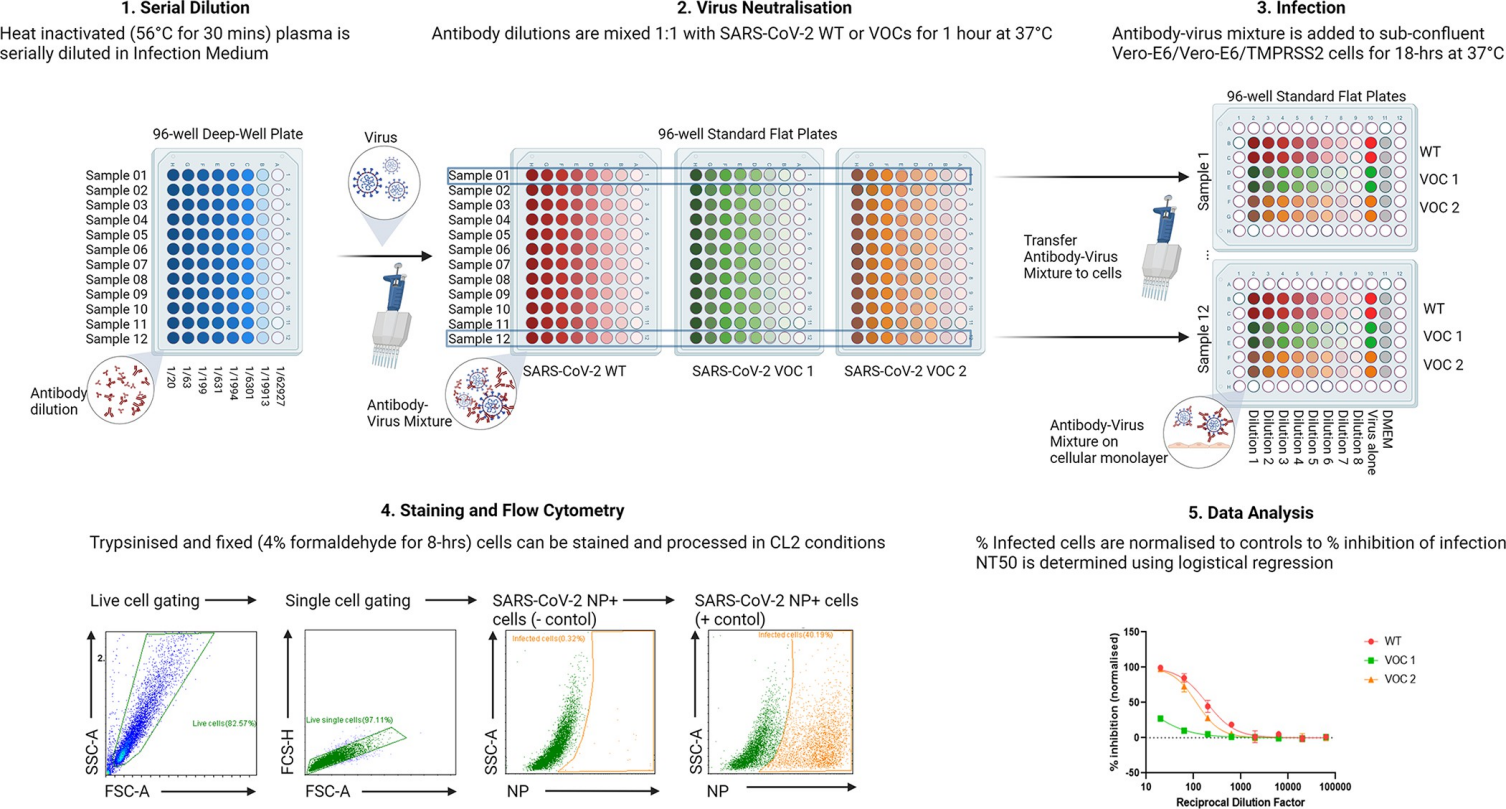
SARS-CoV-2 flow based micro-neutralisation assay workflow. 1. Heat inactivated (30 mins at 56°C) human plasma samples are serially diluted (8-point, half-log from 1:20) in infection medium, in 96-well deep well plates. 2. Viral neutralisation is carried in standard 96-well plates, where each antibody dilution is co-incubated with WT SARS-CoV-2 or a Variant of Concern (VOC) for 60 mins at 37°C. 3. Infection of confluent Vero-E6 or Vero-E6/TMPRSS2 cells with antibody-virus mixture is carried out in 96-well plates for 18h at 37°C. Each plate can test one plasma sample against three SARS-CoV-2 variants in parallel, in duplicates, including controls (virus alone and infection medium alone) or three samples with only one variant. 4. Cells are trypsinised, fixed (4% formaldehyde), permeabilised and stained for intracellular SARS-CoV-2 NP. Infection is measured using Flow Cytometry, first gating the live, single cell population, and then NP negative and positive populations. 5. Flow cytometry data is analysed to determine the percentage of infected (NP+) cells per well and then the percentage of inhibition can be determined by normalising each dilution to the positive and negative controls. NT50 can be determined from the inhibition curve, as the dilution factor resulting in 50% inhibition of infection. Created with BioRender.com.

Here we aimed to develop a medium-throughput, rapid-turnaround live-virus Neutralisation Assay, to enable screening of large clinical cohorts for COVID-19 vaccine studies, or monoclonal antibody studies. The assay involves 5 steps (Fig 1). Firstly, plasma from convalescent or vaccinated individuals is heat inactivated and serially diluted. Secondly, the antibody dilutions are co-incubated with live SARS-CoV-2 (viral neutralisation). Thirdly, the virus/antibody mixture is used to infect cells in culture. Next, cells are trypsinised and fixed, followed by intracellular staining and flow cytometry. Finally, the data is analysed to find the Neutralisation Titre resulting in a 50% inhibition of infection (NT50). The NT50 can then be used to easily compare the neutralisation capacities of human plasma between individuals, before or after vaccination, or against different SARS-CoV-2 variants.

### 1. Flow cytometry-cased micro-NT workflow

**Step 1:** Plasma is heat inactivated for 30 mins at 56°C. This ensures inactivation of the complement system which can trigger cytolysis. For clinical trials, or when multiple experiments are planned per sample, after heat-inactivation the plasma can be aliquoted and stored at -80°C, to avoid multiple freeze-thaw events. Optional: prior to dilution, the samples can be filter-sterilised (0.25μM filter). If working with low volumes, this can be done after diluting the plasma 1/20 in infection medium, and then proceeding with a half-log serial dilution (Fig 1.1), starting at 1:20 and continuing to 1:62927.37. Minimum volume required is 10ul plasma for a test against a single variant, or 30ul against 3 variants (See Box 1 on sample processing).

**Box 1. Maximising sample processing**

The 96-well plate format allows for three samples to be run in parallel, in duplicate, on a single plate against one virus (Fig 1.3), or one sample against three SARS-CoV-2 variants. This allows for an 8-fold serial dilution of the plasma, and positive and negative controls, in duplicate. Only the inner 60-wells of the 96-well plate are used, leaving the outer wells available to be filled with Phosphate Buffered Saline (PBS), limiting evaporation from the wells, and avoiding the ‘edge-effect’.

Plasma samples can be serially diluted using a deep-well 96-well plate, or 48-well plates and then divided between 96-well plates for incubation with SARS-CoV-2 VOCs. If using 48-well plates, an adjustable tip spacing multi-channel makes it easy to transfer from the 48-well format to the 96-well format. The neutralisation takes one hour at 37°C 5% CO_2_. Next, culture medium on the cells is replaced with infection medium (100ul per well) and 100ul virus/plasma is added to the cells, which can easily be done with a standard or automated multi-channel pipette. A single user can comfortably process 12 plates simultaneously in 2 hours of total hands on time, but the total throughput per day will depend on the laboratory needs and capacities of the end user.

Cells are trypsinised and transferred to round-bottom plates for fixation and staining. Transfer efficiency can be increased by using an electronic 1ml multi-channel pipette to serially dispense PBS/PFA across a full plate. The percentage of infected cells is measured directly in 96-well plates through automatic flow-cytometric detection of intracellular SARS-CoV-2 NP. Resuspending the cells in 60ul flow running buffer results in a run-time of 60–80 mins per plate, depending on instrument settings. Of note, this work needs to be performed under Biosafety Level 3 guidelines, in line with established Risk Assessments and Standards Operating Procedures in place in each Containment level 3 facility.

**Step 2:** For viral neutralisation, plasma dilutions are co-incubated with an equal volume (1:1) live SARS-CoV-2 WT or VOCs for 1-hr at 37°C 5% CO_2,_ to facilitate antibody-virus interaction (Fig 1.2).

**Step 3:** Antibody-virus mixture is then added to confluent Vero E6 or Vero E6/TMPRSS2 cells in 96-well plates and the cells are incubated for 18h at 37°C, 5% CO_2_ (Fig 1.3). Controls include wells with no virus (infection medium alone) and virus alone (no plasma). If there is a concern surrounding toxicity from the plasma samples, a plasma control can be used in place of infection medium alone as a negative control to monitor cell viability. In this study, we performed a cell viability assay on Vero E6/TMPRSS2 cells following 18h in culture with secondary standard of COVID-19 convalescent or vaccinated plasma and found negligible impact on cell viability (S1 Fig).

The SARS-CoV-2 variants used are replication competent, live-virus, capable of completing full replication and re-infection cycles, through cell-to-cell contact and also through release of infectious particles into the supernatant. 18h is enough time for the cells to produce viral proteins abundantly, allowing for easy distinction of infected and non-infected cells during analysis, while not providing enough time for viral cytopathic effects to develop. This is important as cells must be intact for subsequent staining and flow-cytometry analysis. See Box 2 for full details of the infectious model.

**Box 2. SARS-CoV-2 infectious model**

The assay was developed with Vero E6 cells, commonly used for SARS-CoV-2 neutralisation assays [37–39] well as with Vero E6/TMPRSS2 cells, as the cellular protease TMPRSS2 is an important factor for SARS-CoV-2 entry, facilitating membrane fusion. However, amplification of SARS-CoV-2 on cells lacking the TMPRSS2 receptor can lead to a well-described cell-line adaption, whereby deletions or mutations in the furin-cleavage site, at the S1/S2 junction, allows the virus to become TMPRSS2-independent, in as few as two passages [40,41]. For this reason, we advise Vero E6/TMPRSS2 as the cellular model of choice for viral isolation and neutralisation assays, though both cell lines remain a common choice [42,43].

When multiple SARS-CoV-2 variants are tested in parallel, the stock viruses are titrated to infect 30–40% of cells after 18h, as measured by flow cytometry. This provides a large enough population of infected cells to observe the neutralisation of the virus across the plasma dilution series, without risking a saturation effect.

**Step 4:** At 18h post-infection, and following trypsinisation, the cells are resuspended in PBS with a final concentration of 4% Formaldehyde to facilitate cell fixation and viral inactivation for a minimum of 30 minutes or according to laboratory specific Risk Assessment (RA) and Standard Operating Procedure (SOP). Once the virus is inactivated, the plates can be transferred to Containment Level 2 facilities to be permeabilised and stained for intracellular SARS-CoV-2 NP. Flow cytometry is then used to measure NP positive cells, as a percentage of the total gated live, single-cell population for each well (Fig 1.4). The gating strategy is described in detail in Box 3.

**Box 3. Flow cytometry staining and analysis strategy**

Here we use intracellular SARS-CoV-2 NP as our target protein. As NP is more conserved than Spike [44], the same antibody can be used to detect WT-B, WT-B.1.177.18, Beta and Omicron-BA.5 variants. There is no homology to cellular proteins that might result in high background staining. Furthermore, as NP is the most abundantly expressed SARS-CoV-2 protein, it can be detected 8h post-infection and is clearly distinguishable from uninfected cells by 18h post-infection, which we found to be the optimal duration of infection for this assay.

Gating cells by Side Scatter (SSC) which indicates granularity, and Forward Scatter (FSC) which indicates size, allows viable cells to be distinguished from dead cells (low FSC, typical SSC) and cellular debris (low FSC and low SSC). Single cells can be separated from doublets or clumped cells by gating the FSC-Area against FSC-Height, which should be a linear relationship. We can then plot the live, single cells against FITC, which for us is the channel used to detect NP staining. Using our negative control wells (cells cultured in infection medium only, no virus) we gate to the immediate right of the cell population. As these cells should not contain any NP, <3% of cells should be considered positive. The same gate should then be applied to all wells. The positive controls (virus in infection medium, no plasma) should be checked to ensure the level of infection is within working range (30–40%).

**Step 5:** Flow cytometry data can be exported as a CSV file for data analysis (Fig 1.5). Mean and standard deviation can be calculated for each well. Wells where there are <1000 live, single events do not have enough cells to be representative of the population and should be removed from the analysis. Background level in the negative control wells should be subtracted from all the readings. The data can then be presented as a percentage of inhibition of infection, where the negative controls have 100% inhibition, and the positive controls have 0% inhibition. The NT50 can then be determined by a non-linear regression (inhibitor versus normalised response).

### 2. Micro-NT produced comparable neutralisation titres to PRNT

**Fig 2:**
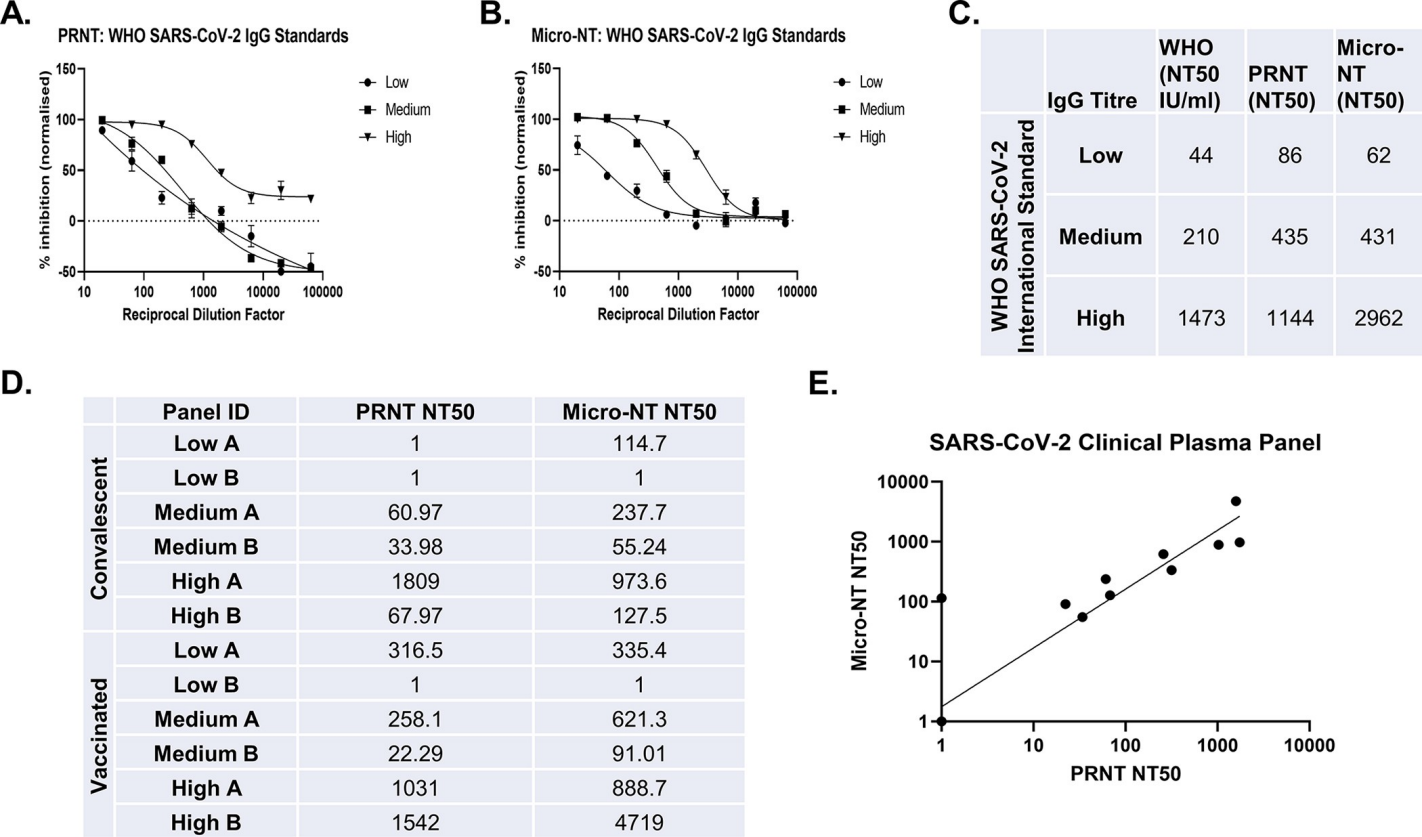
Validation of Flow-Based Micro-NT using the First WHO International Standard for anti-SARS-CoV-2 immunoglobulin and Secondary Standards, (A-B) SARS-CoV-2 (WT-B) was neutralised for 1 hour with an 8-point, half-log serial dilution of WHO SARS-CoV-2 low, medium or high Titre IgG. (A) The antibody-virus mixture was then plated onto Vero E6 cells for 90 mins before overlay (1% CMC) was added and incubated for 96-hours. Plaques were quantified and the inhibition of infection is presented as a percentage, relative to the virus-only control wells. Graph shows the mean and standard deviation of technical duplicates. (B) In parallel, the virus-antibody mixture was added onto Vero E6 cells for 18h before cells were trypsinised, fixed and permeabilised, and stained for the SARS-CoV-2 NP. Percentage of infected cells was determined by flow cytometry analysis. Inhibition of infection is presented as percentage, relative to the virus-only control wells. Graph shows the mean and standard deviation of technical duplicates. (C) Table shows the NT50 values obtained for the three WHO international standards on PRNT and Micro-NT, as well as the NT50 value provided by WHO for each sample in IUs. (D and E) 12 secondary standards were tested in technical duplicates using PRNT and Micro-NT against WT-B.1.177.18 on Vero E6/TMPRSS2 cells. NT50s were determined for each sample (D) and the correlation was plotted using non-linear regression (log-log line) with GraphPad Prism (E).

We compared the NT50 values obtained using Micro-NT on both Vero E6 and Vero E6/TMPRSS2 cells to PRNT, as this is a gold standard viral neutralisation assay. We used the First WHO International Standard for anti-SARS-CoV-2 immunoglobulin (human), a pool of eleven human plasma samples from convalescent patients with low, medium, or high titre SARS-CoV-2 S IgG, established by the WHO Expert Committee on Biological Standardisation in December 2020, to serve as global reference reagents to enable comparison between NT50 values generated by various neutralisation assays, performed by different laboratories, as well as facilitate the generation of International Units (IU) [45]. Both assays were able to distinguish low, medium, and high IgG titre standards based on their neutralising capacity. The NT50 values were determined for each standard and compared between assays. The NT50 for the low IgG standard was very similar between the two assays 86 for PRNT, versus 62 with Micro-NT (Fig 2A–2C). Both assays produced the same NT50 with the medium IgG standard (435 with PRNT and 431 with Micro-NT). Both assays found a high NT50 with the high IgG standard, with Micro-NT showing greater separation between the medium and high Titre results than the PRNT assay (1144 with PRNT and 2962 with Micro-NT). The fold change between the medium and high results with Micro-NT closely reflected the fold change between the antibody potencies in IU/ml as provided by the WHO (7-fold increase between medium and high IgGs using WHO NT50s and 6.9-fold increase using Micro-NT NT50s).

To complement and expand on this validation strategy, we created our own secondary standards, using pooled clinical plasma samples originating from convalescent and vaccinated individuals to create a panel of 12 secondary standards with high, medium or low neutralisation capacity (Fig 2D). Working with clinical plasma samples means that volumes are often limited so this approach allowed us to create multiple aliquots for larger scale testing.

We ran these 12 secondary standards against WT-B.1.177.18 on Vero E6/TMPRSS2 cells, measuring neutralisation capacity both by PRNT and Micro-NT and were able to identify low, medium and high neutralising samples with both assays. We observed a good concordance across most of the standards tested and moderate correlation between both assays, achieving an R^2^ value of 0.5457 (Fig 2D and 2E).

### 3. Micro-NT has good inter-assay and intra-assay reproducibility

To evaluate the precision of the assay, we tested six secondary standards. The neutralisation titre of each was measured five times on a single plate, and this was repeated across five different days. The NT50 values for each run were determined and the repeatability (intra-assay variation) and the intermediate precision (inter-assay variation) were determined using the template provided by Andreasson and colleagues [46].

We obtained a mean NT50 of 121.4, 249.2 and 1006 with the secondary convalescent standards of low, medium and neutralisation capacity (Table 2). Repeatability was 16.6%, 11% and 12.4% respectively and intermediate precision was 27.5%, 34.5% and 23.3%.

**Table 2:** Reproducibility of Micro-NT 6 secondary standards were run against WT-B.1.177.18 SARS-CoV-2 on Vero E6/TMPRSS2 cells, five times per plate (Row 1–5), on five independent days (Day 1–5). The NT50s determined for each row are provided, as well the mean across all 25 measurements. The standard deviation (S) and co-efficient of variation (%CV) is provided for both the repeatability test (R) and the intermediate-precision (inter-assay) test (RW). The mean repeatability and intermediate precision is provided which an average of the 6 measurements.

								Repeatability		Intermediate Precision	
		Row 1	Row 2	Row 3	Row 4	Row 5	Mean Value	S_R_	% CV_R_	S_RW_	% CV_RW_
**Convalescent Low A**	**Day 1**	155.2	153.3	125.8	179.3	161	121.4	20.2	16.6	33.3	27.5
	**Day 2**	151.1	162.5	135.2	151.3	120.9					
	**Day 3**	107.2	149.1	123.1	117.5	97.35					
	**Day 4**	127.8	97.09	84.97	81	101.5					
	**Day 5**	87.5	132.3	93.49	73	65.43					
**Convalescent Medium A**	**Day 1**	202.9	258.8	263.8	233.1	216.2	249.2	27.5	11	86	34.5
	**Day 2**	168.2	131.1	138.3	172.9	142.1					
	**Day 3**	200.4	220.6	218.4	212.9	219.3					
	**Day 4**	382.1	333.1	399	432.5	320.3					
	**Day 5**	299.3	258.2	264.2	293.3	248.6					
**Convalescent High A**	**Day 1**	1096	1192	1054	1267	1027	1006	124.7	12.4	234.6	23.3
	**Day 2**	890.3	858.3	968.3	871.2	912.9					
	**Day 3**	817.4	808.7	694	823	728.2					
	**Day 4**	967.4	859.5	974.3	891.9	960.3					
	**Day 5**	1580	996.1	1510	1249	1154					
**Vaccinated Low A**	**Day 1**	353.5	498.8	415.1	421.3	361.7	340.4	37.7	11.1	70.7	20.8
	**Day 2**	339.4	395.1	361.8	369.7	381.9					
	**Day 3**	365	355.5	273.5	289	313.2					
	**Day 4**	235	212.8	301.4	227.9	249.1					
	**Day 5**	341.4	372.6	379.6	377.9	343.2					
**Vaccinated Medium A**	**Day 1**	661.8	586.4	622.1	622.2	674.5	632.1	49.8	7.9	58.5	9.3
	**Day 2**	737.1	638.6	709.7	649.4	711.6					
	**Day 3**	609.4	558.9	618.5	612.9	616.5					
	**Day 4**	609.2	541.2	753.4	643.8	662.3					
	**Day 5**	550.9	675.2	555.4	609.4	572.6					
**Vaccinated High A**	**Day 1**	687.5	721	920.9	831.6	698	969.8	114.5	11.8	378.3	39
	**Day 2**	565.7	631.8	773.4	587.8	652.9					
	**Day 3**	1787	1790	1443	1401	1508					
	**Day 4**	848.6	944.7	861.6	932	951.8					
	**Day 5**	1075	874.3	863.9	1032	861.6					
**Mean %**									**11.8**		**25.733**

Similarly, we obtained a mean NT50 of 340.4, 632.1 and 969.8 using our secondary vaccinated standards of low, medium and high neutralisation capacity. Repeatability was 11.1%, 7.9% and 11.8% and intermediate precision was 20.8%, 9.3% and 39% respectively.

Cell based neutralisation assays involve actively replicating cells and virus which introduces inherent biological variation. This complexity means the accepted variation is understood to be higher than for *in vitro* binding assays, and variation of up to 25–30% is considered acceptable [31,47–50]. Our assay had an overall repeatability of 11.8% and an intermediate precision of 25.733% which shows good reproducibility.

### 4. Micro-NT has a broad dynamic range

**Fig 3:**
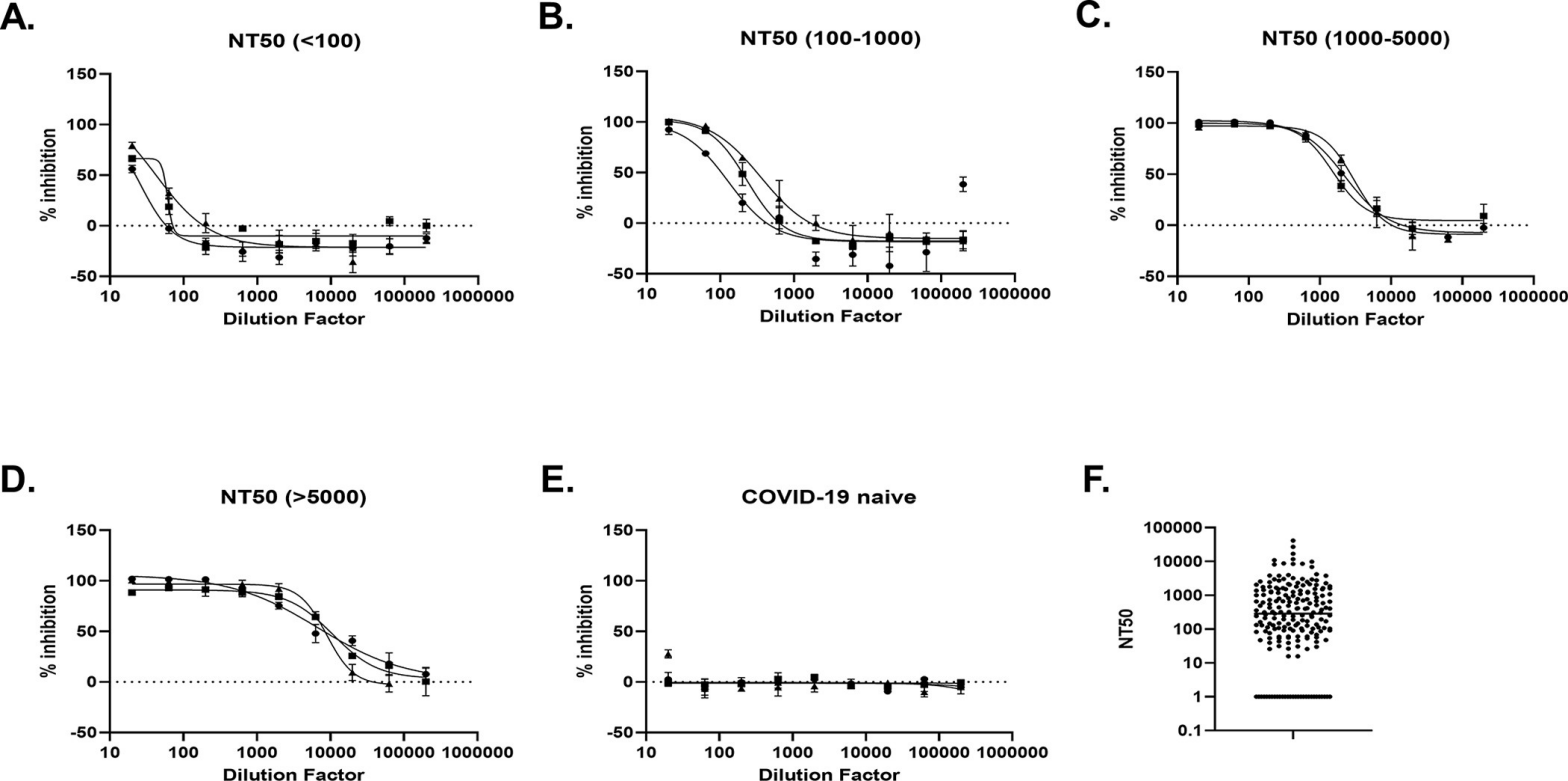
Dynamic range of flow cytometry-based micro-NT. We analysed the NT50s of 201 COVID-19 naïve or convalescent plasma samples, using Micro-NT, against SARS-CoV-2 (WT-B) on Vero E6 for 18h. (A-D) Graphs show neutralising capacity of 3 convalescent plasma samples (P1-P3) across 8-point, half-log serial dilution, representative of samples with 50% Neutralisation Titres (NT50) within a similar range. The percentage of viral inhibition of each dilution was calculated relative to the virus only control. (E) Plasma from COVID-19 naïve individuals showed little or no neutralisation capacity against SARS-CoV-2. (F) Graph shows the full range of NT50 values obtained across the cohort of COVID-19 convalescent plasma samples, measured using Micro-NT.

Across 190 COVID-19 convalescent plasma samples tested, we have found a broad range distribution of neutralising capacities, from 1 to >5000, without saturating the assay. The lower Limit of Detection (LOD) of the assay is an NT50 of 20, corresponding to the initial antibody dilution. Any sample unable to achieve a minimum of 50% inhibition with the initial dilution is given an arbitrary NT50 of 1. An 8-point, half-log dilution from 1/20, to 1/62927, provides an optimal range for measuring neutralising capacity against SARS-CoV-2 from convalescent plasma samples (Fig 3A–3D). Of note, we measured and reported the anti-SARS-CoV-2 IgG titres (Spike S1, Spike S2, Spike RBD and Nucleoprotein) of this convalescent cohort [51] and observed a strong concordance between the NT50 determined using Micro-NT and the IgG titres, with the strongest relationship being anti-RBD (R^2^ of 0.81), in line with published data. In the same report, we described similar correlations between NT50 and anti-RBD across vaccinated (R^2^ of 0.76), and hybrid-immune cohorts (R^2^ of 0.77) [51].

Collectively this work strongly supports that Micro-NT is a robust, highly reproducible assay that has been successfully implemented for the screening of clinical cohorts.

### 5. Micro-NT can be used to measure modulation in neutralising capacity against SARS-CoV-2 Variants of Concern

SARS-CoV-2 VOCs are so named because of a variety of characteristics including increased pathogenicity, increased viral replication capacity, increased infectiousness and/or, of interest to this work, immune escape mutations. Beta was the first VOC described with moderate immune escape, first detected in October 2020, while Omicron BA.5 emerged much later, in April 2022, and as with other Omicron subtypes, displayed significant immune escape due to large numbers of mutations across the Spike protein. As these VOCs are divergent both genetically, chronologically and in their neutralisation capacity, we selected these as representative immune-escape VOCs with which to validate our assay.

**Fig 4:**
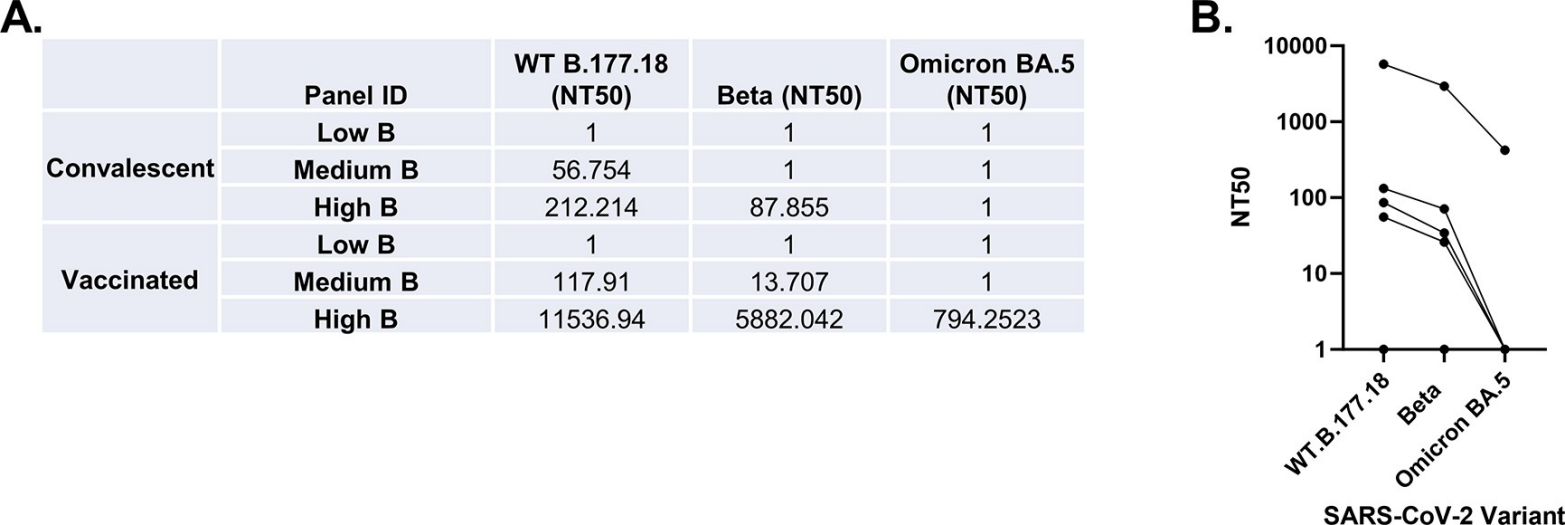
Neutralisation capacity of secondary convalescent and vaccinated standards against SARS-CoV-2 variants of concern measured using micro-NT. Here neutralisation capacity of WT.B-177.18, Beta, and Omicron BA.5 was measured using Micro-NT on Vero E6/TMPRSS2 cells, against six secondary standards. The assays were performed in technical duplicates. (A) NT50 values obtained for each standard against the three SARS-CoV-2 variants. (B) Graph shows the reduction in neutralisation capacity of Beta and Omicron VOCs across each standard tested. Lines represent paired samples.

To examine whether Micro-NT can identify and characterise immune-escape SARS-CoV-2 VOCs, we used three secondary convalescent standards and three secondary vaccinated standards with high, medium and low neutralisation capacities. Convalescent Low B and Vaccinated Low B secondary standards could not neutralise any of the viral isolates tested (Fig 4A and 4B). The remaining four medium and high secondary standards could all neutralise both WT-B.1.177.18 and Beta, although with an average 2.12-fold reduction in neutralisation capacity against Beta. Only Vaccinated High B was able to neutralise Omicron BA.5, though with a 13.6-fold reduction compared to WT.B.1.177.18 and a 6.9-fold reduction compared to Beta. This moderate immune escape elicited by the Beta variant and the significant immune escape by Omicron BA.5 compared to WT SARS-CoV-2 is similar to that reported previously [52,53].

This flow-cytometry based Micro-NT is a medium throughput, reproducible, live SARS-CoV-2 assay, comparable to PRNT but suitable for screening clinical cohorts for NAbs against SARS-CoV-2 variants

### Conclusion

Here we have introduced and presented in detail a new flow-cytometry-based Micro-Neutralisation Assay to quantify the neutralisation capacity of antibodies from plasma. Live SARS-CoV-2 is neutralised by antibodies from convalescent/vaccinated plasma and then used to infect Vero-E6 or Vero-E6/TMPRSS2 cells in a 96-well plate format. After 18h, cells are trypsinised, fixed and stained for intra-cellular SARS-CoV-2 NP. Flow cytometry is then used to quantify the percentage of infected cells per plasma dilution, which allows determination of the NT50 for each plasma sample, against WT SARS-CoV-2 or VOCs.

This approach has been calibrated and validated against the First WHO International Standard for anti-SARS-CoV-2 immunoglobulin, and using secondary standards developed using convalescent and vaccinated pooled clinical plasma samples, against WT and VOCs. We have obtained comparable results to PRNT, on both Vero E6 and Vero E6/TMPRSS2 cell lines with WT SARS-CoV-2.

Relying on visual CPE as a proxy for infection runs a risk of cell death through other causes, e.g. contamination, toxicity or drying being mistaken for viral mediated cytotoxicity. The viral infection here is measured through intracellular staining of the SARS-CoV-2 protein, similar to other established micro-neutralisation assays, which provides a specific indicator of infection [24,28].

Flow cytometry has several advantages as an assay endpoint. Firstly, infection is quantifiable, being able to see the number of infected cells as well as the percentage of the whole population. Secondly, once the plates are stained, a flow cytometer with an automatic plate reader will process all wells without requiring user input, other than set-up and cleaning, limiting hands-on processing time. Furthermore, as cells are fixed prior to staining, flow analysis can take place in CL2 conditions when compact flow cytometers are placed within Biosafety Cabinets to control for risk of aerosol formation. This protocol expands the options to perform live virus neutralising assays to those with access to flow-cytometry facilities, which is becoming more common as labs are investing in this popular and diversely applicable technique.

As with all live virus assays, limitations include access to CL3 facilities, with trained staff to carry out live virus work. Other limitations include a higher volume of viral inoculum being needed to infected 30–40% of infected cells over 18h compared to the inoculum required for PRNT or micro-foci assay, where <1% of cells are infected over the course of the assay. Furthermore we have only carried out the above analysis using human plasma samples, not serum, so further validation would be required to use this approach for serum screening.

While pseudovirus assays performed in CL2 conditions can address some of these limitations, live-virus assays remain the gold standard. New VOCs can be rapidly isolated from nasopharyngeal swabs, and once validated, can be used directly in the assay. By titrating the viruses to infect the same percentage of cells after 18h, neutralisation capacity can be determined against several SARS-CoV-2 variants in parallel, without needing to account for differences in CPE, or duration of time to development of CPE both of which can vary across SARS-CoV-2 variants [54,55].

Flexibility in SARS-CoV-2 variants and antibody sources means the assay is easily adapted to multiple applications. We have used the flow-based Micro-NT to define an RBD titre post-infection/vaccination that correlates with a protective neutralisation titre [51] as well as to identify COVID-19 convalescent individuals as donors for convalescent plasma therapy early in the pandemic [56]. During initial COVID-19 vaccine clinical trials, high infection and hospitalisation rates allowed for collection of data on protection from infection or severe disease [57–60]. Indeed, neutralisation titres post vaccination were found to be directly correlated with vaccine efficacy [61]. However, as vaccine uptake increases, and COVID-19 cases decrease, such clinical endpoints are harder to achieve. Surrogate endpoints, including neutralisation titres will therefore play an important role in measuring vaccine efficacy in next generation vaccine trials [61,62]. VACCELERATE, an EU Consortium is currently co-ordinating clinical trials identifying vaccine efficacy paediatric populations (EudraCT Number: 2021-005043-71) as well as the safety, timing, and efficacy of COVID-19 booster vaccines in the fully vaccinated (EudraCT number 2021-004889-35). The flow-cytometry based micro-neutralisation assay described here was chosen to enable the measurement of NAbs across large numbers of clinical specimens (>400) across multiple SARS-CoV-2 variants.

## Methods

### Cells

Vero E6 (VERO C1008, Vero 76, clone E6, Vero E6), were obtained from ATCC (ATCC CRL-1587) and maintained in Dulbecco’s Modified Eagle’s Medium (DMEM, Thermo Scientific, 61965–026) supplemented with 10% Foetal Bovine Serum (Themo Scientific, 10500–064) (DMEM-10). Vero E6/TMPRSS2 cells (#100978), obtained from the Centre For AIDS Reagents (CFAR) at the National Institute for Biological Standards and Control (NIBSC) [63] were cultured in DMEM supplemented with 10% Foetal Bovine Serum (FBS, DMEM-10) and Geneticin (Thermo Scientific, 10131035) at a concentration of 1mg/ml, at 37°C in 5% CO2. All cell lines were routinely tested for mycoplasma. During the course of infection, cells were incubated in infection medium, DMEM 2% FBS (DMEM-2) supplemented with Penicillin (1U/ml) and Streptomycin (100ug/ml) (Sigma-Aldrich, P4333) and amphotericin B (0.5ug/ml) (Thermo Fisher Scientific, 15290018) at 37°C, 5% CO_2_ in a humidified incubator.

### Human plasma samples

Plasma derived from ethylenediamintetraacetic acid (EDTA) anticoagulated whole blood was collected from COVID-19 RT-qPCR positive and negative participants of the All-Ireland Infectious Disease (AIID) Cohort [64]. For this analysis we included either individuals with polymerase chain reaction (PCR)-confirmed COVID-19 (convalescent), documented vaccination with 2 doses of mRNA-1273, BNT162b2 or ChAdOx1-S, at least 14 days from the second dose (vaccinated), or individuals without COVID-19 or vaccination recruited with available bio-banked plasma dated no later than July 2019, prior to the onset of the COVID-19 pandemic (COVID-19 naïve). All participants demographics and characteristics together with measurement of anti-Spike and anti-Nucleoprotein IgGs values have been previously described in [51]. A total of 190 convalescent plasma samples from COVID-19 patients were tested in this study. 11 non-COVID-19 plasma samples, collected prior to the COVID-19 pandemic and negative for anti-SARS-CoV-2 antibodies were included as negative controls. Samples were stored at -80°C before use. All human plasma samples were heat-inactivated at 56°C for 30 minutes before testing. First WHO International Standard for anti-SARS-CoV-2 immunoglobulin, human (NIBSC code: 20/266) is a pool of eleven human plasma from convalescent patients and was established in December 2020 by the WHO Expert Committee on Biological Standardization. Secondary standards were composed of twelve pooled plasma samples from the AIID convalescent or vaccinated cohort with low, medium or high neutralisation capacity. Secondary standards were prepared in single use aliquots and stored at -80°C prior to use. The AIID Study and these analyses were approved by the relevant local and national research ethics committees [51].

### SARS-CoV-2 clinical isolates

All work with live SARS-CoV-2 was carried out in Containment Level 3 laboratory under Biosafety Level 3 guidelines. WT Spike, Pango lineage B (WT-B) SARS-CoV-2 clinical isolate 2019-nCoV/Italy-INMI1, Clade V, Passage 4 [65] was obtained from the European Virus Archive goes Global (EVAg, Spallanzani Institute, Rome) and amplified on Vero E6 cells (Passage 6) (sequencing information is available in S1 Table). WT SARS-CoV-2 with D614G substitution (Pango lineage B.1.177.18 (WT-B.1.177.18), GenBank accession ON350866, Passage 2), Beta (Pango lineage B.1.351, GenBank accession ON350868, Passage 2) and Omicron (Pango lineage BA.5 (Omicron-BA.5), GenBank accession OP508004, Passage 1) clinical isolates were isolated from SARS-CoV-2 positive nasopharyngeal swabs from the AIID cohort. Supernatant was filter sterilised (0.2nm filter) and diluted 1:1 with infection medium. Confluent Vero E6/TMPRSS2 cells were incubated with the virus in a T12.5 flask until 50% cell death was observed using a light microscope. The supernatant and cells were transferred to a confluent T175, with infection medium. Cells were cultured until 50% cell death was observed. The supernatant was collected and centrifuged at 4000xg for 5 minutes, aliquoted and stored at -80°C. The titre of the virus stocks was determined by plaque assay. Each isolate was sequenced before use. Here viral RNA was isolated using the Qiagen QIAamp Viral RNA mini kit according to manufacturer’s instructions. Sanger sequencing was then carried out by Eurofins Genomics. WT-B had a titre of 5x10^4^ PFU/ml WT-B.1.177.18 had a titre of 2.6x10^6^ PFU per ml, Beta had a titre of 1.2x10^6^ PFU/ml and Omicron-BA.5 had a titre of 2.2x10^6^ PFU/ml.

### Flow cytometry-based micro-neutralisation assay

#### Viral neutralisation

Heat-inactivated plasma samples were diluted with a half-log, 8-point serial dilution, from a starting dilution of 1:20 in infection medium. SARS-CoV-2 isolates were titrated to infect 30–40% of Vero E6/Vero E6/TMPRSS2 cells 18-hr post infection as determined by flow cytometry analyses of SARS-CoV-2 Nucleoprotein positive cells. Viral isolates were diluted in infection medium and co-incubated with the plasma dilutions in a 1:1 ratio for 1 hour at 37°C, 5% CO_2._

#### Infection

Vero E6 or Vero E6/TMPRSS2 cells were plated (2.5x10^4^) the day before use in 100ul culture medium, in clear flat-bottom 96-well plates (Sarstedt, 83.3924). The cells were incubated overnight at 37°C 5% CO_2_ to reach 100% confluency at time of infection. Post-neutralisation, supernatant was removed from the cells and replaced with 100ul infection medium and 100ul virus/plasma mix, in triplicates or duplicates. Positive controls (virus alone) and negative controls (infection medium alone) were included in each plate. Cells were incubated for 18 hours at 37°C 5% CO_2_.

#### Cell collection

Supernatant was discarded and cells were rinsed with 100ul PBS. 25ul Trypsin-EDTA (Gibco, # 25300054) was added to the cells at 37°C until single-cell suspension was obtained. Cells were resuspended in 75ul PBS and transferred to a round-bottom 96-well plate (Sarstedt, 83.3925500) with 100ul 8% formaldehyde solution (Sigma Aldrich, F8775) to achieve a final concentration of 4%. Cells were fixed in the dark at room temperature overnight.

#### Cell staining

All steps-post fixation were carried out in Biosafety Level 2 laboratory in Class 2 Biosafety Cabinets. All centrifugation was carried out at 4000xg for 5 minutes (Heraeus Megafuge 16R, Thermo Scientific), with 96-well plates contained within sealed buckets (Thermo Scientific, #75003625). The supernatant was removed, and cells were permeabilised with Perm/Wash Buffer (BD, 554723) according to manufacturer’s instructions, which was maintained throughout antibody staining. Intracellular SARS-CoV-2 Nucleoprotein (NP) staining was performed with SARS/SARS-CoV-2 Nucleocapsid Monoclonal Antibody (E16C) (1:100 dilution, Invitrogen, MA1-7403), goat anti-mouse IgG2b-FITC (1:500 dilution, Santa Cruz Biotechnology, SC-2080).

#### Flow cytometry

Cells were resuspended in 60ul PBS-EDTA-2% for flow cytometry analysis (Beckman Coulter CytoFlex or CytoFlex S). Forward and Side-Scatter gates were used to exclude debris from intact cells. Cells were then gated using Forward Scatter Area where single cells were gated based on linearity between Area and Height, excluding doublets. % Infected cells of the single cell population were determined using the negative control wells to set the boundary of the negatively staining populating in the FITC-channel (Blue 488nm laser, 525/40 filter). Gating was performed using CytExpert software (version 2.4.0.28, Beckman Coulter).

#### Analysis

Wells with <1000 single events were excluded from analysis. Positive controls(virus only, no plasma) should be in the working range (30–40% infected) while negative controls (infection medium only) had to be <3% or the plate would be excluded from analysis. The mean percentage of infected cells for each plasma dilution was calculated, and this result was normalised using the control wells, where 0% viral inhibition was equal to the positive control and 100% viral inhibition was equal to the negative control. The plasma dilution resulting in a 50% reduction in infection (NT50) was determined using non-linear regression, (inhibitor v normalised response with GraphPad Prism (Version 9.3.1).

### Plaque Reduction Neutralisation Test (PRNT)

Vero E6 cells or Vero E6/TMPRSS2 cells were plated (4.2x10^5^) in 1ml DMEM-10 in clear flat-bottom 12-well plates. The cells were incubated overnight at 37°C 5% CO_2_ to reach 100% confluency at time of infection. The First WHO International Standard for anti-SARS-CoV-2 immunoglobulin, with low, medium and high titre IgG or secondary standards were diluted with a half-log, 8-point serial dilution, from a starting dilution of 1 in 20 in infection medium. SARS-CoV-2 (WT-B) or WT-B.1.177.18 were diluted in complete infection medium to a concentration of 100 Plaque Forming Units (PFU) per 100ul and co-incubated with the standard dilutions in a 1:1 ratio for 1 hour at 37°C 5% CO_2_. Supernatant was removed from the cells and 200ul plasma/virus was added to each well. Plate was gently shaken to ensure the surface of the well was evenly coated. The plates were incubated at 37°C 5% CO_2_ for 90 mins, with shaking every 10 mins. 2ml complete infection medium with 1% carboxymethylcellulose (CMC, Sigma #C5013) was added to each well. The plates were incubated for 48-96-hours until plaques became visible. 2ml 8% formaldehyde solution was added directly to each well to a final concentration of 4% and the plates were fixed overnight in the dark at room temperature. The supernatant was removed from the cells and the cells washed 3 times in ddH_2_O until all CMC was removed. Crystal violet (Sigma-Aldrich, #HT90132) 0.5% diluted in 25% MeOH, 75% H_2_O, was added to just cover the surface of the well, incubated for 15 mins at room temperature, then removed with 2 washes in ddH_2_O. Plates were allowed to fully dry before being photographed. A modified ImageJ script [66] was used to count the plaques per well and the duplicates were averaged and normalised to the positive control (virus only). The % inhibition per dilution was analysed with a non-linear regression (variable slope) on GraphPad Prism to determine the NT50.

### Cell viability assay

To measure the impact of plasma on cell viability, secondary standards panel A were diluted in infection medium with an 8-point, half-log dilution series starting at 1:20. The plasma standards were incubated on confluent Vero E6/TMPRSS2 cells in the absence of SARS-CoV-2 and incubated for at 37°C 5% CO_2_ in a humidified incubator. After 18h, cell viability was determined using Cell Counting Kit 8 (CCK8) (Abcam #ab228554) according to manufacturer’s instructions. Following a 2-hr incubation period, the absorbance was read using a microplate reader at 450nm. The percentage of cell viability was determined relative to the infection medium alone (no plasma) control. Each dilution was tested in technical duplicates, in two independent experiments.

## Supporting information

S1 Fig Plasma samples from a panel of convalescent or vaccinated samples, with low, medium or high neutralising capacity were serially diluted in Infection Medium and co-incubated with confluent Vero E6/TMPRSS2 cells for 18h. Cell viability was measured using Cell Counting Kit-8 reagent. Percentage viable cells was determined by normalising all samples to an Infection Medium only control. Samples were tested in technical duplicates. Data shown represents mean and standard deviation of two independent experiments.
(TIF)

S1 Table Sequencing information on SARS-CoV-2 WT-B passage 6, after amplification on Vero E6 cells. (XLSX)

## Abbreviations

S: Spike
NP: Nucleoprotein
NAbs: Neutralising Antibodies
RBD: Receptor Binding Domain
ACE-2: Angiotensin-Converting Enzyme 2
WT: Wild-Type
VOC: Variant of Concern
PRNT: Plaque Reduction Neutralisation Test
CPE: Cytopathic Effects
NT50: 50% Neutralisation Titre
