# Supplementary figures and images for: Development of a novel medium throughput flow-cytometry based micro-neutralisation test for SARS-CoV-2 with applications in clinical vaccine trials and antibody screening
Source: PLoS One. 2023 Nov 30;18(11):e0294262. doi: 10.1371/journal.pone.0294262 (PMC10688860; doi:10.1371/journal.pone.0294262)

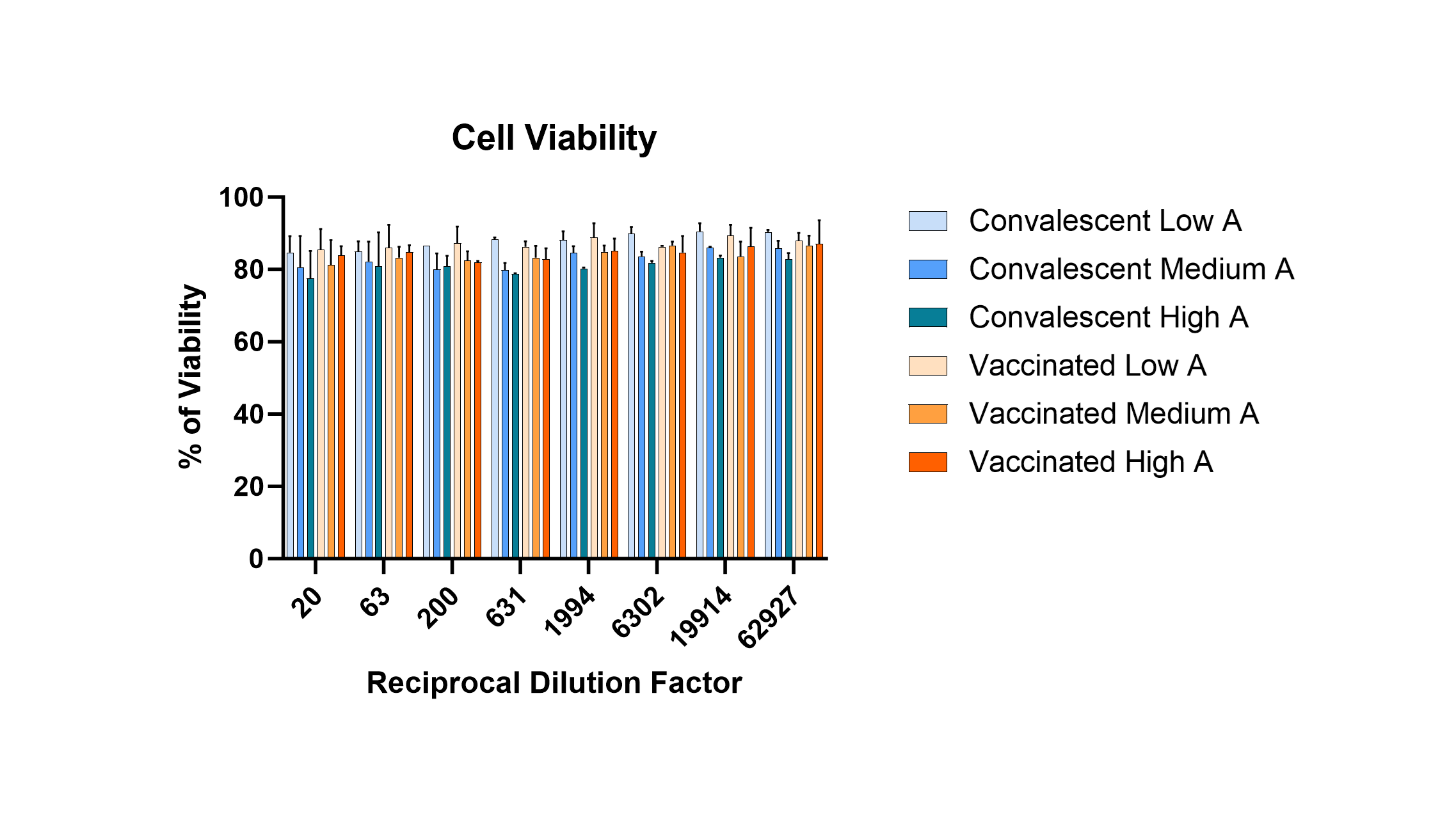

Supplement: S1 Fig — Cell viability was measured using Cell Counting Kit-8 reagent. Percentage viable cells was determined by normalising all samples to an Infection Medium only control. Samples were tested in technical duplicates. Data shown represents mean and standard deviation of two independent experiments. (TIF) [file pone.0294262.s001.tif]
